# Acknowledgement of manuscript reviewers 2021

**DOI:** 10.18332/ejm/145904

**Published:** 2022-01-21

**Authors:** Victoria G. Vivilaki

**Affiliations:** 1 Department of Midwifery, School of Health and Care Sciences, University of West Attica, Athens, Greece

## CONTRIBUTING REVIEWERS

The editors of European Journal of Midwifery would like to thank all our reviewers who have contributed to the journal in Volume 5 (2021).

**Ababor, Sabit**

Ethiopia

**Adams, Crystal**

United States

**Akın, Bihter**

Turkey

**Aksoy-Derya, Yeşim**

Turkey

**Aktas, Songul**

Turkey

**Alalaf, Shahla**

Iraq

**Almasidou, Maria**

Greece

**Altiparmak, Sümeyye**

Turkey

**Ansell, Lesley**

New Zealand

**Auxier, Jennifer**

Finland

**Bączek, Grażyna**

Poland

**Bains, Sukhjeet**

Norway

**Baran, Joanna**

Poland

**Barasinski, Chloé**

France

**Barimani, Mia**

Sweden

**Barrett, Geraldine**

United Kingdom

**Bartlett, Rebeccah**

Australia

**Baş, Nazan**

Turkey

**Baskaya, Yasemin**

Turkey

**Basnet, Til Bahadur**

Nepal

**Bedru-Nasir, Beshir**

Ethiopia

**Benahmed, Nadia**

Belgium

**Berta, Marta**

Sweden

**Berterö, Carina**

Sweden

**Biesty, Linda**

Ireland

**Blanc, Julie**

France

**Bloxsome, Dianne**

Australia

**Bobevski, Irene**

Australia

**Bolman, Maya**

United States

**Bradfield, Zoe**

Australia

**Bradford, Billie**

New Zealand

**Brekalo, Maja**

Croatia

**Can, Hafize**

Turkey

**Carlsson, Ing-Marie**

Sweden

**Carlton, Jill**

United Kingdom

**Carpenter, Jane**

United Kingdom

**Carroll, Lorraine**

Ireland

**Casey, Deborah (Debbie)**

United Kingdom

**Channons, Sue**

United Kingdom

**Chen, Shu-Wen**

Australia

**Çift, Tayfur**

Turkey

**Clarkson, Gina**

United States

**Cohen, Wayne**

United States

**Dadras, Omid**

Iran

**Dahl, Bente**

Norway

**de Castro Bussadori, Jamile**

Brazil

**Dean, Sue**

Australia

**Demissie, Tadesse**

Ethiopia

**Di Marco, Lionel**

France

**Donati, Serena**

Italy

**Donnellan-Fernandez, Roslyn**

Australia

**Ducarme, Guillaume**

Canada

**Emelonye, Abigail**

Uganda

**Erickson, Elise**

United States

**Erkal-Aksoy, Yasemin**

Turkey

**Escañuela-Sánchez, Tamara**

Ireland

**Evensen, Ann**

United States

**Fellmeth, Gracia**

United Kingdom

**Fontein-Kuipers, Yvonne**

Netherlands

**Franková, Věra**

Czech Republic

**Frederiksen, Yoon**

Denmark

**Gebriné, Éles**

Hungary

**Ghaffar, Abdul**

Thailand

**Giaxi, Paraskevi**

Greece

**Gitsels-van der Wal, Janneke**

Netherlands

**Gonzalez-Díaz, Enrique**

Spain

**Greene, Richard**

Ireland

**Grekin, Rebecca**

United States

**Guangzhou, Ouyang**

China

**Güney, Esra**

Turkey

**Hakala, Mervi**

Finland

**Haslam, Michael**

United Kingdom

**Hodgson, Suzanne**

United Kingdom

**Homer, Caroline**

Australia

**Huang, Jing**

China

**Ireland, Sarah**

Australia

**Kaçar, Nükhet**

Turkey

**Karaçam, Zekiye**

Turkey

**Karakoç, Hediye**

Turkey

**Keedle, Hazel**

Australia

**Kerr, Margaret**

United States

**Khatri, Resham**

Nepal

**Kivlighan, Katie**

United States

**Koç, Emine**

Turkey

**Kool, Liesbeth**

Netherlands

**Kumar Das, Manoja**

India

**Kurtz Landy, Christine**

Canada

**Kurz, Ella**

Australia

**Le Lagadec, Marie**

Australia

**Le Ray, Camille**

France

**Leap, Nicky**

Australia

**Li, Shelly-Anne**

Canada

**Li, Zhao**

China

**Liepinaitienė, Alina**

Lithuania

**Lindberg, Marie**

Norway

**Lisi, Cosima**

Portugal

**Londero, Ambrogio**

Italy

**López-Gimeno, Encarnación**

Spain

**Lukasse, Mirjam**

Norway

**Maeda, Eri**

Japan

**Mallen-Perez, Laura**

Spain

**Maude, Robyn**

New Zealand

**Maxwell, Clare**

United Kingdom

**McConville, Fran**

Switzerland

**Mena-Tudela, Desirée**

Spain

**Minooee, Sonia**

Australia

**Mittal, Suneeta**

India

**Mivšek, A. Polona**

Slovenia

**Montanari-Vergallo, Gianluca**

Italy

**Moore, Zena**

Ireland

**Moran, Emma**

Ireland

**Morin, Christine**

France

**Mortensen, Berit**

Norway

**Musie, Maurine**

South Africa

**Młynarska, Agnieszka**

Poland

**Navarro-Prado, Silvia**

Spain

**Nergis, Meryem**

Turkey

**Nespoli, Antonella**

Italy

**Neter, Efrat**

Israel

**Nikitara, Katerina**

Greece

**Nomura, Roseli**

Brazil

**Nunes Ribeiro, Caique**

Brazil

**O’Brien, Denise**

Ireland

**O’Connell, Rhona**

Ireland

**O’Connor, Meaghan**

United States

**Oflu, Ayse**

Turkey

**Ojeda, Felipe**

Spain

**Okonofua, Friday**

Nigeria

**Omer, Sumaira**

China

**Origlia Ikhilor, Paola**

Switzerland

**Overbeck, Gritt**

Denmark

**Pace-Parascandalo, Rita**

Malta

**Pandita, Aakash**

India

**Papadopoulos, Irena**

United Kingdom

**Paplinskie, Stephanie**

Canada

**Paramashanti, Bunga**

Indonesia

**Parodi, Arianna**

Italy

**Paulos, Kebreab**

Ethiopia

**Pay, Aase Serine Devold**

Norway

**Pekcan, Nuriye**

Turkey

**Phipps, Hala**

Australia

**Probst-Marmier, Isabelle**

Switzerland

**Prosen, Mirko**

Slovenia

**Pulgar, Victor M.**

United States

**Riaz, Muhammad**

United Kingdom

**Rodriguez-Coll, Pablo**

Spain

**Roy, Manas**

India

**Sahlin-Ulfsdottir, Hannah**

Sweden

**Schauberger, Charles**

United States

**Shinohara, Eriko**

Japan

**Shishehgar, Sara**

Iran

**Siristatidis, Charalampos**

Greece

**Sisman, Yagmur**

Denmark

**Spencer, Rebecca**

United Kingdom

**Svalenius, Elizabeth**

Sweden

**Terzi, Banu**

Turkey

**Thapa, Shyam**

Nepal

**Thomas, Grace**

United Kingdom

**Thomas, Mary-Powel**

United States

**Toker, Eylem**

Turkey

**Tran Luy, Marie**

France

**Tsakiridis, Ioannis**

Greece

**Tudose, Melania**

Romania

**Tunde-Byass, Modupe**

Canada

**Turan, Zekiye**

Turkey

**Uchmanowicz, Izabella**

Poland

**Velagic, Minela**

Bosnia and Herzegovina

**Vermeulen, Joeri**

Belgium

**Viirman, Frida**

Sweden

**Vismara, Laura**

Italy

**Værland, Inger**

Norway

**Walker, Shawn**

United Kingdom

**Webb, Rebecca**

United Kingdom

**Whitburn, Laura**

Australia

**Wildgaard, Kim**

Denmark

**Wilson, R. Douglas**

Canada

**Wynter, Karen**

Australia

**Yamamoto, Shelby**

Canada

**Younas, Ahtisham**

Canada

**Yurtsal, Zeliha**

Turkey

**Zaami, Simona**

Italy

**Zeitlin, Jennifer**

France

**Zhao, Yun**

China

**Zimmo, Kaled**

Norway

**Zimmo, Mohammed**

Norway

**Zivoder, Ivana**

Croatia

**Zubaran, Carlos**

Brazil

**Žutić, Maja**

Croatia

**Žvanut, Boštjan**

Slovenia

